# A magnetic pulse does not affect free-flight navigation behaviour of a medium-distance songbird migrant in spring

**DOI:** 10.1242/jeb.244473

**Published:** 2022-10-03

**Authors:** Thiemo Karwinkel, Michael Winklhofer, Lars Erik Janner, Vera Brust, Ommo Hüppop, Franz Bairlein, Heiko Schmaljohann

**Affiliations:** ^1^ Institute of Avian Research ‘Vogelwarte Helgoland’, An der Vogelwarte 21, 26386 Wilhelmshaven, Germany; ^2^ Institute for Biology and Environmental Sciences, Carl von Ossietzky University of Oldenburg, Carl-von-Ossietzky-Straße 9-11, 26129 Oldenburg, Germany; ^3^ Research Center for Neurosensory Sciences, Carl von Ossietzky University Oldenburg, Carl-von-Ossietzky-Straße 9-11, 26129 Oldenburg, Germany; ^4^ Institute for Biochemistry and Biology, University of Potsdam, Maulbeerallee 1, 14469 Potsdam, Germany; ^5^ Max Planck Institute of Animal Behavior, Am Obstberg 1, 78315 Radolfzell, Germany

**Keywords:** Bird migration, Magnetic map, Magnetic-particle-based sensor, Magnetic pulse, Magnetoreception, Navigation

## Abstract

Current evidence suggests that migratory animals extract map information from the geomagnetic field for true navigation. The sensory basis underlying this feat is elusive, but presumably involves magnetic particles. A common experimental manipulation procedure consists of pre-treating animals with a magnetic pulse, with the aim of re-magnetising particles to alter the internal representation of the external field prior to a navigation task. Although pulsing provoked deflected bearings in caged songbirds, analogous studies with free-flying songbirds yielded inconsistent results. Here, we pulsed European robins (*Erithacus rubecula*) at an offshore stopover site during spring migration and monitored their free-flight behaviour with a regional-scale network of radio-receiving stations. We found no pulse effect on departure probability, nocturnal departure timing departure direction or consistency of flight direction. This suggests either no use of the geomagnetic map by our birds, or that magnetic pulses do not affect the sensory system underlying geomagnetic map detection.

## Teaser

**Summary:** Magnetic pulse pre-treatment disturbed geomagnetic navigation in caged birds. However, our free-flying songbirds show no effect, questioning the effect of the pulse on or usage of geomagnetic maps.

## INTRODUCTION

Migratory songbirds navigate to familiar breeding and wintering sites with fascinating accuracy and precision, even over distances of several thousands of kilometres (e.g. Salewski et al., 2000; Holland, 2014). One cue that plays a major role in this navigational task is the Earth's magnetic field. Through systematic changes in inclination, declination and total intensity, the Earth's magnetic field forms spatial gradients around the globe (Skiles, 1985) that could be used as a geomagnetic map. Thus, many studies on migrating birds have hypothesised that experienced birds use such a map to perform true navigation, defined as the ability to return to a familiar migratory destination from an unfamiliar location (reviewed in Holland, 2014; Heyers et al., 2017). How birds detect geomagnetic information is still unknown, but is widely believed to be based on small magnetic particles innervated by sensory nerve endings of the trigeminal nerve (Kishkinev et al., 2013; Beason and Semm, 1996). Although we still lack direct anatomical evidence for the postulated magnetic particles (Curdt et al., 2022), their existence is supported by a number of behavioural studies that pre-treated birds with a strong magnetic pulse (e.g. Wiltschko and Wiltschko, 1995; Holland and Helm, 2013). The pulse is aimed at re-magnetising (Wiltschko et al., 1994) or re-arranging magnetic particles in the receptor (Davila et al., 2005), producing an altered sensory output to the unchanged ambient magnetic field. Thus, the magnetic pulse leads to an altered internal representation of the geomagnetic field for the bird. Under the magnetic map hypothesis, the pulsed animal would interpret such an altered magnetic field percept as a different location than present or as a corrupted signal. Indeed, pulsed songbirds mostly showed deflected orientations, not only when tested in artificial orientation cages (Emlen funnels; e.g. Wiltschko and Wiltschko, 1995; Beason et al., 1995; Wiltschko et al., 1998) but also when departure directions were measured in free flight (Holland, 2010; Holland and Helm, 2013). Thereby, the magnetic pulse does not affect the inclination compass of the birds, which is most likely based on a radical-pair-based mechanism containing no magnetic material (reviewed in Hore and Mouritsen, 2016). Moreover, behavioural evidence suggests that a magnetic pulse affects only adult migrants but not juveniles (Holland and Helm, 2013; Munro et al., 1997). Juvenile birds rely on their innate inclination compass during their inaugural migration, but have no navigational map. Therefore, the results of the pulse studies are consistent with a magnetic-particle-based sensor in a geomagnetic map navigation context.

Using a regional high-throughput tracking system with unprecedented spatiotemporal resolution, we recently performed a pulse study comprising 140 individuals of a long-distance migrant songbird, the northern wheatear (*Oenanthe oenanthe*, hereafter wheatear), on the offshore island of Helgoland in the German Bight during their autumn stopover (Karwinkel et al., 2022). To our surprise, pulsed birds and control birds turned out to be statistically indistinguishable in all migration-related behavioural decisions we had monitored, i.e. departure directions, departure probability, nocturnal departure timing and consistency in flight direction over 50–100 km (Karwinkel et al., 2022). Taking all previous results together, the most parsimonious explanation for the absence of a pulse effect is the dispensability of magnetic map cues for the wheatear, at least at this offshore location. After all, the stopover site was still several thousands of kilometres away from the migratory destination in Africa (Schmaljohann et al., 2016), so magnetic map factors may not be essential for determining the flight direction at this particular part of the route. This rationale is supported by a recent ring-recovery study presenting correlative evidence that migratory birds use magnetic inclination angles as a cue when closing in on their target region (Wynn et al., 2022). Further, evidence from displaced adult migratory gulls suggests that a navigational response may not always be shown immediately, but becomes more pronounced when birds approach their migratory destination (Wikelski et al., 2015; cf. Alerstam, 2001).

We consequently expanded our study to further explore the role of distance to the migratory destination on magnetic map navigation and repeated our pulsing study with the same methodology on a short- to medium-distance migrant, the European robin (*Erithacus rubecula*, hereafter robin). During spring stopover on Helgoland, robins are much closer to their migratory destination (some 50 to 1250 km), in this case to their breeding sites (Dierschke et al., 2011), compared with wheatears in our former study, whose destination was their African wintering grounds (Karwinkel et al., 2022). Importantly, in an independently conducted study, free-flying robins have been reported to be affected by magnetic pulse pre-treatment on spring migration (Holland, 2010). However, evidence to date was based on a sample size of only six treated and 13 control robins and a relatively short tracking range (maximum ca. 5.5 km) at a mainland location. Moreover, their experimental procedure did not allow for the control of physiological and environmental conditions *a priori*. Despite showing consistency in wind conditions and migratory readiness *post hoc*, experimental birds were treated later within the year than controls (fig. 2 in Holland, 2010).

Here, we investigated the response to the magnetic pulse of robins during spring migration on four migratory traits: departure probability, departure timing, departure direction and consistency in flight direction for the first 50–100 km after departure. We expected that pulsing would show an effect on how birds extract and interpret geomagnetic map information (cf. Holland, 2010), which in turn would affect their decision of whether to depart, and if so, when and in what direction.

## MATERIALS AND METHODS

### Ethics

All work was conducted under the permission by the Ministry of Energy, Agriculture, the Environment, Nature and Digitalisation of the federal state Schleswig-Holstein, Germany, under permission number V 244–16840/2019(41-4/19).

### Study site

**Fig. 1.**
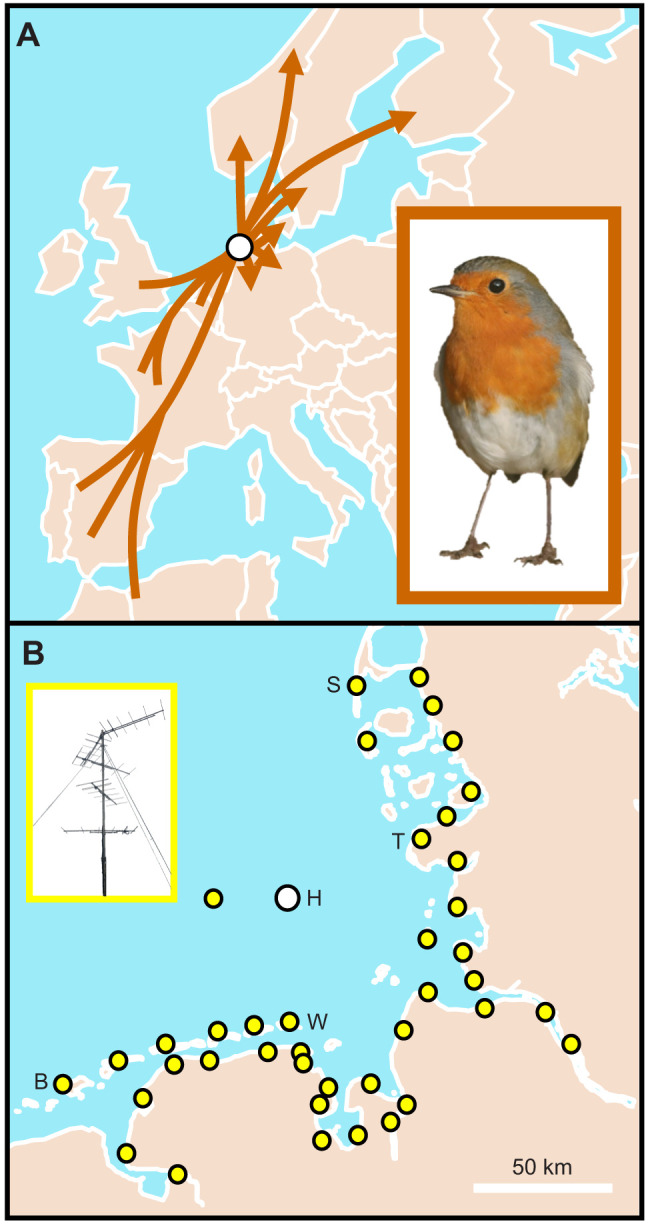
**Study area in the context of European robin (*Erithacus rubecula*) spring migration and network of radio-receiving stations.** (A) Estimated migration routes of European robins (inset) passing Helgoland on spring migration, based on ring recoveries (Dierschke et al., 2011; Bairlein et al., 2014). (B) Network of radio-receiving stations in the German Bight, with every dot representing a radio-receiving station (example shown in the inset). H, Helgoland; B, Borkum; W, Wangerooge; T, Tümlauer Koog; S, Sylt.

The experiment was performed on Helgoland (54°11′N, 07°53′E), which is a small (approximately 1 km²) island in the North Sea (Fig. 1). The closest shorelines are on Wangerooge Island in the south (44 km) and at St Peter-Ording (48 km; near Tümlauer Koog) in the east-northeast (Fig. 1B). The shoreline of the German Bight encompasses Helgoland in a section from about 17 deg (Sylt Island; 72 km) to 232 deg (Borkum Island; 104 km; Fig. 1B). Otherwise, Helgoland is surrounded by open sea, with the Norwegian coast approximately 425 km to the north and the British coast approximately 500 km to the west (Fig. 1A).

### Study species

During spring migration, robins regularly stopover on the island, with up to 200 individuals per day during peak passage from the end of March to the end of April (Dierschke et al., 2011). Robins are solitary night migrants, and thus their migratory behaviour is independent of conspecifics (Schmaljohann and Klinner, 2020; Packmor et al., 2020; Dorka, 1966). According to ring recoveries, robins passing Helgoland in spring migrate to northern Germany, southern Scandinavia and the Baltic region (Fig. 1A; Dierschke et al., 2011; Bairlein et al., 2014). The proposed breeding area is therefore approximately 50 to 1250 km away from our study side. Radio-tracking data of migrating robins from Helgoland show a mean departure direction of 90 deg in spring (Klinner, 2020). Robins are irregular breeders with a maximum of 1–2 pairs on Helgoland (Dierschke et al., 2011) and no breeding record in summer 2021 (J. Dierschke, pers. comm.). Consequently, all robins included in our experiments were migrants. We caught robins with Helgoland funnel traps and mist nets between 24 March and 22 April 2021 throughout the day. Birds were aged according to Svensson (1992) and Jenni and Winkler (2020). Only second-calendar-year birds entered the experiment to exclude any potential age effects. Maximum wing chord of each individual was measured to the nearest 0.5 mm (Svensson, 1992).

### Bird housing

After catching, birds were immediately transferred to individual cages (40×30 cm base, 40 cm high) within the island station of the Institute of Avian Research ‘Vogelwarte Helgoland’, where they received artificial light in the natural day:night rhythm. Birds were provided with *ad libitum* access to water and food (mealworms, *Tenebrio molitor*, supplemented with fat-food, Fett-Alleinfutter Typ II grün, Claus GmbH, Germany) to allow for accumulation of fat, i.e. accumulating energy stores. By that, we ensured that all birds carried sufficient and comparable energy stores important for high motivation to resume migration, as many studies have shown a significant positive correlation between energy stores and departure probability (Schmaljohann et al., 2022; Schmaljohann and Eikenaar, 2017; Klinner et al., 2020; Eikenaar et al., 2021).

### Experimental treatment with a magnetic pulse

As night-migratory songbirds most likely decide to resume migration several hours before sunset (Eikenaar et al., 2020), we conducted the experiment 6 h before sunset to provide pulsed birds with sufficient time to include the altered geomagnetic map percept into their migratory decision-making process. Pulsed birds that did not directly depart in the first night after treatment can be assumed to still have perceived an altered geomagnetic field over the following days, because former studies demonstrated effects for approximately 10 days after magnetic pulsing (Wiltschko et al., 2007; Holland and Helm, 2013). We conducted the experiments only on days with favourable migration conditions (wind <8 m s^−1^, no rain) to minimise weather-dependent effects on migratory decisions (Erni et al., 2002; Packmor et al., 2020). On these days, at approximately 7 h before sunset, we divided the housed robins into two equally sized groups. To calculate the birds' energy stores (Kelsey et al., 2020), we weighed them to the nearest 0.1 g and estimated their muscle score (Bairlein, 1994). We found no difference in energy stores between the treatment and control groups (Welch’s two-sample *t*-test: *t*_78_=−0.852, *P*=0.315), which implies comparable physiological states between the groups.

**Fig. 2.**
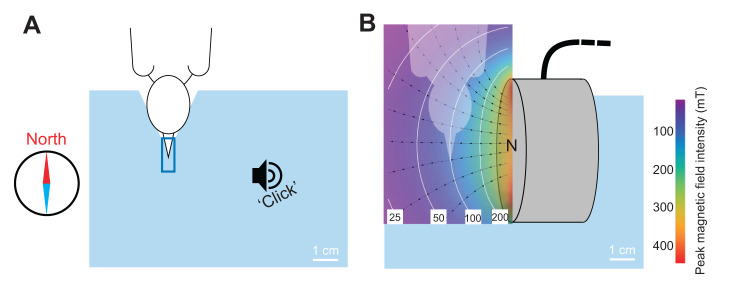
**Control and experimental treatment with a magnetic pulse.** (A) While hand-holding the bird, its head was gently fixed against an indentation on a styrofoam block, with its beak inserted in a small plastic tube (blue rectangle). Control birds experienced just a short ‘click’ sound. (B) Birds in the experimental group were similarly hand-held, but experienced a magnetic pulse from a coil (grey cylinder). White lines on the heat map show isolines of peak magnetic field intensity. Black lines are selected magnetic field lines. Figure adapted from Karwinkel et al. (2022).

Birds in the treatment group were exposed to a magnetic pulse of 0.1 T peak strength using the pulse geometry and protocol detailed in Karwinkel et al. (2022). In brief, the pulse was delivered with a small coil placed on the side of the beak and focused onto the middle part of the beak (Fig. 2), where the putative magnetic-particle-based sensor is thought to be located (Heyers et al., 2017). In the moment the pulse was fired, the birds were hand-held and their heads were fixed in a short piece of tubing (Fig. 2A) to ensure that the application geometry of the pulse was the same for each bird (Fig. 2). The head of the bird pointed southwards and the magnetic field lines of the pulse were aligned perpendicularly to the bird's head, with the magnetic North pole pointing towards the bird (Fig. 2B). This perpendicular pulse field geometry was found to cause a significantly larger deflection of homing pigeons on the day of treatment compared with the axial pulse geometry (Beason et al., 1997).

The magnetic pulse was produced by a coil with a 5 cm diameter and 15×15 windings of 1 mm copper wire connected to a small generator (‘Beck-Pulser’, magnetic pulse generator, Ing. Büro L. Albrecht, Heist, Germany). The magnetic field reached its maximum after 1.5 ms and decayed within 8 ms (see fig. 2C,D in Karwinkel et al., 2022). We ensured functionality of the pulse application at every experimental day by controlling peak pulse intensity with a magnetometer (Gaußmeter HGM09s, MAGSYS Magnet Systeme GmbH, Dortmund, Germany). The control group experienced the same handling procedure, but the magnetic pulse was replaced by a short ‘click’ sound produced by tapping a finger on the structure, resembling the sound of the magnetic pulser at firing (Fig. 2A). After either treatment, we attached a radio-transmitter to the bird and released it before processing the next bird. We performed this experimental procedure on 31 March 2021 with 12 birds, on 20 April 2021 with 44 birds and on 25 April 2021 with 24 birds (*n*_total_=80). Each time, half of the birds entered the control group and the other half the treatment group.

### Radio-tracking free-flight behaviour

Birds were fitted with radio-transmitters (NTQB-2, Lotek Wireless Inc., Canada; burst intervals between 2.3 and 5.3 s) via individually adjusted leg-loop harnesses (Naef-Daenzer, 2007). The total mass of the radio-transmitters including harness was approximately 0.31 g, which did not exceed 2.0% of the bird's body mass (median: 1.8%) and is therefore well below the 3–5% recommended threshold (Kenward, 2001). To track the behaviour of the birds in free flight, we used an automated radio-receiving system on Helgoland (Müller et al., 2018a; Karwinkel et al., 2022). This system consists of four SensorGnome receivers (www.sensorgnome.org) at three sites on Helgoland, connected to 16 radially aligned antennas (6-element Yagi antennas, Vårgårda Radio AB, Sweden), ensuring a radial resolution of 22.5 deg (Fig. S1A). Further, the German Bight is equipped with 40 comparable automated radio-receiving stations (Fig. 1B) (Brust et al., 2019; Karwinkel et al., 2022), which allowed us to track the birds passing the shoreline of the German Bight after departure from Helgoland. All stations are part of the Motus Wildlife Tracking System (see http://www.motus.org and Taylor et al., 2017). From the radio-tracking data, as received from Motus, we determined (1) whether the bird stayed or departed in the first night after the treatment (departure probability), (2) the departure timing within the night, (3) the departure direction from Helgoland and (4) the consistency of this direction towards the shoreline (Fig. S1B), using an algorithm written by the authors, in a replicable and blinded approach to avoid any observer bias. For further details about the radio tracking data analysis, see Supplentary Materials and Methods or Müller et al. (2018b) and Packmor et al. (2020).

### Weather data

We considered weather parameters at two time points. First, weather parameters were assigned to the night after release at 200 min after sunset, which was the median departure timing in a former study with free-flying robins on Helgoland (Packmor et al., 2020), and second, for the individual time of actual departure. Precipitation (mm) and cloud cover (eighths) were provided by a local weather station on the island operated by the German Weather Service (DWD). Airspeed flow assistance (hereafter wind assistance) (m s^−1^) for a flight direction of 41 deg (Klinner, 2020) was calculated using NCEP reanalysis data (NOAA, Boulder, CO, USA, https://rda.ucar.edu/datasets/ds090.0/; Kemp et al., 2012a,b). Wind assistance has the advantage of including multiple relevant wind parameters (e.g. side winds, tailwind component) as well as the bird’s own airspeed in a single unit.

### Statistics

All statistical analyses were performed using R 4.0.3 statistical software (https://www.r-project.org/). To assess whether our treatment affected the birds' departure probability in the first night (departing or not departing, binomial), we ran a generalised linear model (GLM) including the following predictor variables: treatment condition (experiment/control; categorical), wind assistance (continuous), cloud cover (categorical) and energy stores (continuous). Here, weather parameters were considered from 200 min after sunset for the night after release (see section above). We *z*-transformed all numeric variables. Because model assumptions were violated, as detected by goodness-of-fit plots (Korner-Nievergelt et al., 2015), and as we did not find a transformation to solve this problem, we had to discard the model and ultimately used a chi-square test to assess the potential difference in the departure probability between the control and experimental groups.

Because nights were getting shorter during the course of the experiment (night duration of first experiment: 664 min; night duration of last experiment: 558 min), to determine an individual's departure timing, we calculated the proportion of night at departure for each individual separately. To do so, an individual's departure time (minutes after sunset) was divided by the night length on the night of departure (min). To explain variation in the departure timing (log-transformed), we applied a linear model (LM) including the following predictor variables: treatment condition (experiment/control; categorical), wind assistance (continuous), cloud cover (categorical), day of year of release (Julian Day, continuous) and energy stores (continuous), and all two-way interactions with the treatment condition (all numerical variables *z*-transformed). Weather parameters and day of year were assigned for the individual departure timing. Predictor variables were found to be linearly independent (VIF<2; http://CRAN.R-project.org/package=usdm). Because none of the two-way interactions came out as significant, they were removed from the final model. The residual analyses did not indicate violation of model assumptions (see R code in the Supplementary Materials and Methods and Table S2 for details).

To compare the circular variables (departure direction, consistency in flight direction) between the groups, we applied circular statistics using the R packages ‘CircStats’ (http://www.CRAN.R-project.org/package=CircStats) and ‘circular’ (http://www.CRAN.R-project.org/package=circular). Owing to the arrangement of the radio-tracking antennas on Helgoland (Fig. S1A), our directional data were grouped. As the Mardia–Watson–Wheeler test randomly breaks ties (in our case the groups), we repeated the test 10,000 times to exclude any bias owing to random tie-breaking, and then provided the median of the test parameters (see R code in the Supplementary Materials and Methods for details).

## RESULTS AND DISCUSSION

### Effects on the four migratory traits

**Fig. 3.**
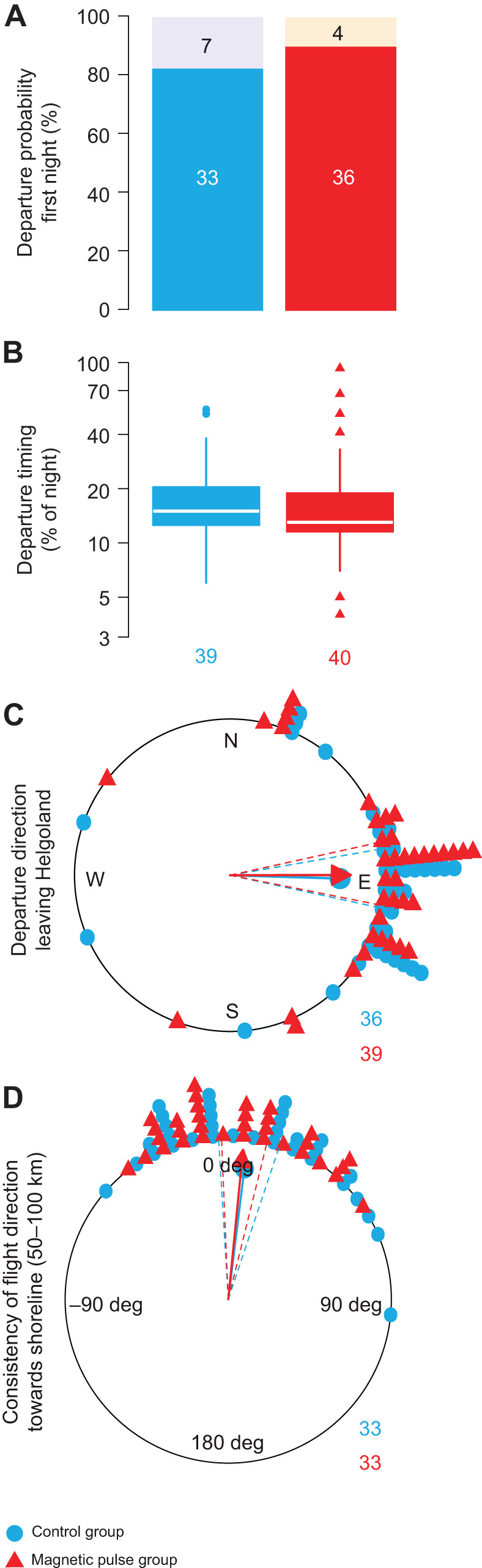
**Effect of a magnetic pulse on four migratory traits in free-flying European robins (*Erithacus rubecula*) during spring migration.** Numbers indicate sample sizes per group, representing individual birds. Sample sizes decreased in sequence as not every trait could be characterised for every bird (for details, see Materials and Methods). (A) Departure probability as percentage of birds departing on the first night after the release from Helgoland (white numbers, lower bar solid colour) or staying for the first night (black numbers, upper bar transparent colour). (B) Nocturnal departure timing as percentage of the night at departure for all birds. Control: 1st quartile: 13%, median: 15%, 3rd quartile: 21%, range: 6–55%. Treatment: 1st quartile: 12%, median: 13%, 3rd quartile: 19%, range: 4–93%. (C) Initial departure direction from Helgoland. Control: Rayleigh test: *r*=0.77, *P*<0.001, mean: 92 deg. Treatment: Rayleigh test: *r*=0.78, *P*<0.001, mean: 90 deg. (D) Consistency of flight direction after departure from Helgoland until passage at the shoreline of the German Bight (50–100 km), given as the directional deviation between departure direction from Helgoland and passage location site on the shoreline (Fig. 1B, see Materials and Methods for more details). Control: Rayleigh test: *r*=0.86, *P*<0.001, mean: 7 deg. Treatment: Rayleigh test: *r*=0.92, *P*<0.001, mean: 6 deg. Data points in the circular plots are shifted slightly off-centre by <5 deg to better distinguish the data of the corresponding groups.

Departure probability was 83% in the control group (33 out of 40 robins departed in the first night after release) and 90% in the magnetic-pulse treated group (36 out of 40 departed in the first night; Fig. 3A, Table S1). Our results thus fail to demonstrate an effect of the magnetic pulse, supposed to alter geomagnetic map information, on the motivation to migrate as departure probability between the groups did not differ (Pearson's χ²-test: χ²_1_=0.42, *P*=0.52). A former study demonstrated that artificially altered geomagnetic field information on caged birds indeed affected nocturnal migratory restlessness (Bulte et al., 2017), which is a direct proxy for the departure probability in the wild (Eikenaar et al., 2014). However, whereas Bulte et al. (2017) altered the geomagnetic field consistent with a meaningful spatiotemporal occurrence of the study species, we potentially corrupted the putative magnetic particle-based sensor of the robins with a magnetic pulse in an unknown way. This makes it impossible to predict whether the field percept from the hypothesised sensor corresponds to a meaningful location to our birds. Nevertheless, the study of Bulte et al. (2017) shows that changes in geomagnetic cues affect the motivation to depart in caged birds, but our results and those of Karwinkel et al. (2022) did not verify this for free-flying songbirds.

Regarding nocturnal departure timing, robins in the control group had a median departure after 15% of the night length had elapsed, and robins of the magnetic-pulse treated group after 13% (Fig. 3B). We found no effects of the treatment condition (*P*=0.64; Fig. 3B) or day of year (*P*=0.64) on nocturnal departure timing (Table 1). Increasing energy stores (*P*=0.03) and wind assistance (*P*<0.001), but decreasing cloud cover (*P*=0.002), advanced nocturnal departure timing (Table 1), which is consistent with the natural departure behaviour of free-flying birds, i.e. without caging and feeding (e.g. Packmor et al., 2020). This reflects that we can confidently assume that our experimental procedure does not alter the natural migration behaviour of robins in this study.

**Table 1.**
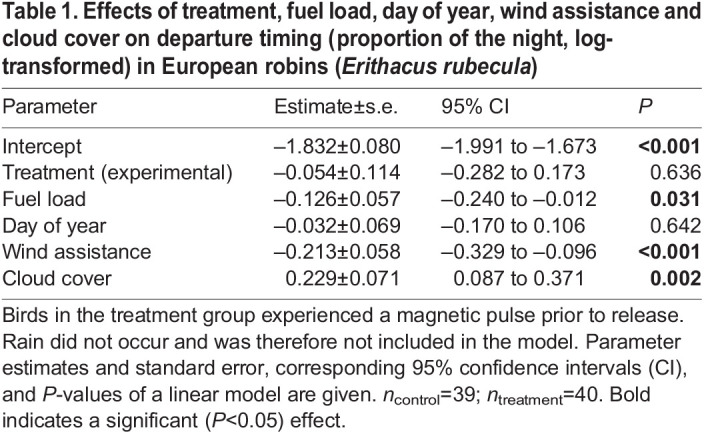
Effects of treatment, fuel load, day of year, wind assistance and cloud cover on departure timing (proportion of the night, log-transformed) in European robins (*Erithacus rubecula*)

Departure directions from Helgoland of both groups were significantly oriented to the east from Helgoland (Fig. 3C) and did not differ from each other (repeated Mardia–Watson–Wheeler test: *W*_2_=0.33, *P*=0.85). The robins consistently maintained their flight direction from Helgoland of approximately 50–100 km towards the shoreline (Fig. 3D) and their consistency did not differ between groups (Watson–Williams test: *F*_1,64_=0.04, *P*=0.842). Consequently, departure directions from Helgoland are representative of the sustained migratory flight direction during the night. Therefore, we are convinced that there was no biologically significant effect of the magnetic pulse on departure direction. This is in stark contrast to the findings of Holland (2010), who found a 90 deg clockwise shift in departure direction after a magnetic pulse applied to the same species, also during spring.

### Is geomagnetic map information dispensable for migratory birds?

Former magnetic pulse studies (e.g. Wiltschko and Wiltschko, 1995; Holland and Helm, 2013) suggested that birds use geomagnetic map information obtained via a magnetic-particle-based sensor. This is in contrast to our findings where, despite large statistical power to resolve effects if present, pulse pre-treatment did not significantly affect migratory behaviour and decisions, neither in robins as reported here, nor in our parallel study on wheatears (Karwinkel et al., 2022). We can safely assume that our pulse was strong enough to affect the putative magnetite-based magnetoreceptor and to provoke altered magnetic field perceptions persisting over the observation time (see Karwinkel et al., 2022).

Provided that magnetic particles are the sensory basis for obtaining geomagnetic map information, a key requirement for the magnetic pulse to have an effect on migratory decisions is that birds indeed rely on this information. In former studies, this reliance was taken for granted, although it was mostly unknown before the experiments had been performed. The challenge is that we do not know when, where and how often migratory songbirds integrate geomagnetic map information in their migratory decision-making process, e.g. potentially also upon arrival at a stopover site. This integration might be highly context-dependent and vary with factors such as distance to the migratory destination, the availability of other navigation cues such as landmarks, or even the physiological state of the bird. Therefore, it remains speculative whether and, if so, which factors would affect birds differently at our experimental site on Helgoland compared with other sites, e.g. in Holland (2010) and Holland and Helm (2013), who found the expected pulse effect in free-flying birds.

A pulse may affect only those birds that do not actively notice a corrupted geomagnetic map reading that is inconsistent with other navigation or even orientation cues. This situation is most likely to apply to an indoor experiment where birds have no other cues available than the Earth's magnetic field to determine their location and direction towards the migratory destination. In contrast, free-flying birds, as in our experiment, have access to all available environmental cues, e.g. landmarks, which might be conveniently used instead of the geomagnetic map information for the migratory decisions. Therefore, the difference in the availability of cues may at best explain the discrepancy between corresponding cage studies (e.g. Wiltschko and Wiltschko, 1995; Beason et al., 1995) and our free-flight studies (present study; Karwinkel et al., 2022), but falls short of reconciling our negative results with the former studies that obtained clear pulse effects in free-flying birds (Holland, 2010; Holland and Helm, 2013).

### Do birds need magnetic particles for obtaining geomagnetic map information?

Because we do not know whether geomagnetic map information is dispensable for the birds' migratory decisions at our test site, a pulse study with more diagnostic power would ideally be conducted at a site at which we know that birds rely on geomagnetic map cues for these decisions. Sites deemed most suitable are those where evidence for magnetic map navigation was obtained from real or virtual translocation experiments, e.g. in Chernetsov et al. (2008) or Kishkinev et al. (2015). If birds pulsed in such a location head into different directions than control birds, the proposed experiment would clearly link the geomagnetic map to the magnetic pulse and strongly suggest a sensory system based on magnetic particles. Even if such a pulse treatment would not produce an observable effect at all, one cannot necessarily dismiss the magnetic particle hypothesis given the pulse effects observed in some previous indoor studies (Wiltschko and Wiltschko, 1995; Beason et al., 1995; Wiltschko et al., 1998). Nonetheless, a negative result would invite an alternative hypothesis, namely that geomagnetic map information is probably obtained with the inclination compass (Wynn et al., 2022), i.e. on the basis of the radical-pair mechanism.

Of the three geophysical gradients of the Earth's magnetic field (inclination, declination and total intensity), the radical-pair-based mechanism may be able to detect at least inclination and declination (declination only in combination with a celestial compass). Whereas there is direct evidence for the use of declination (Chernetsov et al., 2017), and partly also for inclination (Wiltschko and Wiltschko, 1992; Wynn et al., 2022) as geomagnetic cues, convincing evidence for the biological importance of magnetic intensity for songbird navigation is currently lacking. In this light, our results question whether treating migratory birds with a magnetic pulse affects the appropriate sensory system crucial to geomagnetic map navigation in free-flying birds.

## Supplementary Material

10.1242/jexbio.244473_sup1 Supplementary information

## References

[JEB244473C1] Alerstam, T. (2001). Detours in bird migration. *J. Theor. Biol.* 209, 319-331. 10.1006/jtbi.2001.2266 11312592

[JEB244473C2] Bairlein, F. (1994). *Manual of Field Methods. European-African Songbird Migration*. Wilhelmshaven, Germany: Institut für Vogelforschung “Vogelwarte Helgoland”.

[JEB244473C3] Bairlein, F., Dierschke, J., Dierschke, V., Salewski, V., Geiter, O., Hüppop, K., Köppen, U. and Fiedler, W. (2014). *Atlas des Vogelzugs. Ringfunde deutscher Brut- und Gastvögel*. Wiebelsheim: AULA-Verlag.

[JEB244473C4] Beason, R. C. and Semm, P. (1996). Does the avian ophthalmic nerve carry magnetic navigational information? *J. Exp. Biol.* 199, 1241-1244. 10.1242/jeb.199.5.1241 9319100

[JEB244473C5] Beason, R. C., Dussourd, N. and Deutschlander, M. E. (1995). Behavioural evidence for the use of magnetic material in magnetoreception by a migratory bird. *J. Exp. Biol.* 198, 141-146. 10.1242/jeb.198.1.141 9317510

[JEB244473C6] Beason, R. C., Wiltschko, R. and Wiltschko, W. (1997). Pigeon homing: effects of magnetic pulses on initial orientation. *The Auk* 114, 405-415. 10.2307/4089242

[JEB244473C7] Brust, V., Michalik, B. and Hüppop, O. (2019). To cross or not to cross: thrushes at the German North Sea coast adapt flight and routing to wind conditions in autumn. *Mov. Ecol.* 7, 32. 10.1186/s40462-019-0173-5 31695918 PMC6824093

[JEB244473C8] Bulte, M., Heyers, D., Mouritsen, H. and Bairlein, F. (2017). Geomagnetic information modulates nocturnal migratory restlessness but not fueling in a long distance migratory songbird. *J. Avian Biol.* 48, 75-82. 10.1111/jav.01285

[JEB244473C9] Chernetsov, N., Kishkinev, D. and Mouritsen, H. (2008). A long-distance avian migrant compensates for longitudinal displacement during spring migration. *Curr. Biol.* 18, 188-190. 10.1016/j.cub.2008.01.018 18249113

[JEB244473C10] Chernetsov, N., Pakhomov, A., Kobylkov, D., Kishkinev, D., Holland, R. A. and Mouritsen, H. (2017). Migratory Eurasian reed warblers can use magnetic declination to solve the longitude problem. *Curr. Biol.* 27, 2647-51.e2. 28823677 10.1016/j.cub.2017.07.024

[JEB244473C11] Curdt, F., Haase, K., Ziegenbalg, L., Greb, H., Heyers, D. and Winklhofer, M. (2022). Prussian blue technique is prone to yield false negative results in magnetoreception research. *Sci. Rep.* 12, 1-16. 10.1038/s41598-021-99269-x 35614116 PMC9132912

[JEB244473C54] Davila, A. F., Winklhofer, M., Shcherbakov, V. P. and Petersen, N. (2005). Magnetic pulse affects a putative magnetoreceptor mechanism. *Biophys. J.* 89, 56-63. 10.1529/biophysj.104.049346 15863473 PMC1366555

[JEB244473C12] Dierschke, J., Dierschke, V., Hüppop, K., Hüppop, O. and Jachmann, K. F. (2011). *Die Vogelwelt der Insel Helgoland*. Helgoland: OAG Helgoland.

[JEB244473C13] Dorka, V. (1966). Das Jahres- und tageszeitliche Zugmuster von Kurz- und Langstreckenziehern nach Beobachtungen auf den Alpenpässen Cou/Bretolet (Wallis). *Ornithol. Beob.* 63, 165-223.

[JEB244473C14] Eikenaar, C., Karwinkel, T. and Hessler, S. (2021). Changes in fat mass affect the motivation to migrate in northern wheatears. *J. Avian Biol.* 52. 10.1111/jav.02736

[JEB244473C15] Eikenaar, C., Klinner, T., Szostek, K. L. and Bairlein, F. (2014). Migratory restlessness in captive individuals predicts actual departure in the wild. *Biol. Lett.* 10, 20140154. 10.1098/rsbl.2014.0154 24718095 PMC4013702

[JEB244473C16] Eikenaar, C., Schaefer, J., Hessler, S., Packmor, F. and Schmaljohann, H. (2020). Diel variation in corticosterone and departure decision making in migrating birds. *Horm. Behav.* 122, 104746. 10.1016/j.yhbeh.2020.104746 32217064

[JEB244473C17] Erni, B., Liechti, F., Underhill, L. G. and Bruderer, B. (2002). Wind and rain govern the intensity of nocturnal bird migration in central Europe - a log-linear regression analysis. *Ardea* 90, 155-166.

[JEB244473C18] Heyers, D., Elbers, D., Bulte, M., Bairlein, F. and Mouritsen, H. (2017). The magnetic map sense and its use in fine-tuning the migration programme of birds. *J. Comp. Physiol. A Neuroethol. Sens. Neural Behav. Physiol.* 203, 491-497. 10.1007/s00359-017-1164-x 28365788

[JEB244473C19] Holland, R. A. (2010). Differential effects of magnetic pulses on the orientation of naturally migrating birds. *J. R Soc. Interface* 7, 1617-1625. 10.1098/rsif.2010.0159 20453067 PMC2988258

[JEB244473C20] Holland, R. A. (2014). True navigation in birds: from quantum physics to global migration. *J. Zool.* 293, 1-15. 10.1111/jzo.12107

[JEB244473C21] Holland, R. A. and Helm, B. (2013). A strong magnetic pulse affects the precision of departure direction of naturally migrating adult but not juvenile birds. *J. R Soc. Interface* 10, 20121047. 10.1098/rsif.2012.1047 23389901 PMC3627120

[JEB244473C22] Hore, P. J. and Mouritsen, H. (2016). The radical-pair mechanism of magnetoreception. *Annu. Rev. Biophys.* 45, 299-344. 10.1146/annurev-biophys-032116-094545 27216936

[JEB244473C23] Jenni, L. and Winkler, R. (2020). *Moult and Aging of European Passerines*. London: Bloomsbury.

[JEB244473C24] Karwinkel, T., Winklhofer, M., Christoph, P., Allenstein, D., Hüppop, O., Brust, V., Bairlein, F. and Schmaljohann, H. (2022). No apparent effect of a magnetic pulse on free-flight behaviour in northern wheatears (*Oenanthe oenanthe*) at a stopover site. *J. R. Soc. Interface* 19, 20210805. 10.1098/rsif.2021.0805 35167773 PMC8847002

[JEB244473C25] Kelsey, N., Schmaljohann, H. and Bairlein, F. (2020). A handy way to estimate lean body mass and fuel load from wing length: a quantitative magnetic resonance data approach. *Ring. Migr.* 34, 2-24.

[JEB244473C26] Kemp, M. U., Shamoun-Baranes, J., van Loon, E. E., McLaren, J. D., Dokter, A. M. and Bouten, W. (2012a). Quantifying flow-assistance and implications for movement research. *J. Theor. Biol.* 308, 56-67. 10.1016/j.jtbi.2012.05.026 22683380

[JEB244473C27] Kemp, M. U., van Loon, E., Shamoun-Baranes, J. and Bouten, W. (2012b). RNCEP: global weather and climate data at your fingertips. *Methods Ecol. Evol.* 3, 65-70. 10.1111/j.2041-210X.2011.00138.x

[JEB244473C28] Kenward, R. E. (2001). *A Manual for Wildlife Radio Tagging*. London, San Diego: Academic Press.

[JEB244473C29] Kishkinev, D., Chernetsov, N., Heyers, D. and Mouritsen, H. (2013). Migratory reed warblers need intact trigeminal nerves to correct for a 1,000 km eastward displacement. *PLoS One* 8, e65847. 10.1371/journal.pone.0065847 23840374 PMC3694148

[JEB244473C30] Kishkinev, D., Chernetsov, N., Pakhomov, A., Heyers, D. and Mouritsen, H. (2015). Eurasian reed warblers compensate for virtual magnetic displacement. *Curr. Biol.* 25, R822-R824. 10.1016/j.cub.2015.08.012 26439333

[JEB244473C31] Klinner, T. (2020). What's the difference? Migration behaviour of long- and medium-distance migratory songbirds. *PhD thesis*, University of Oldenburg, Germany.

[JEB244473C32] Klinner, T., Buddemeier, J., Bairlein, F. and Schmaljohann, H. (2020). Decision-making in migratory birds at stopover: an interplay of energy stores and feeding conditions. *Behav. Ecol. Sociobiol.* 74, 1-14. 10.1007/s00265-019-2784-7

[JEB244473C33] Korner-Nievergelt, F., Roth, T., von Felten, S., Guélat, J., Almasi, B. and Korner-Nievergelt, P. (2015). *Bayesian data analysis in ecology using linear models with R, BUGS, and Stan*. London: Elsevier.

[JEB244473C34] Müller, F., Eikenaar, C., Crysler, Z. J., Taylor, P. D. and Schmaljohann, H. (2018a). Nocturnal departure timing in songbirds facing distinct migratory challenges. *J. Anim. Ecol.* 87, 1102-1115. 10.1111/1365-2656.12821 29504627

[JEB244473C35] Müller, F., Rüppel, G. and Schmaljohann, H. (2018b). Does the length of the night affect the timing of nocturnal departures in a migratory songbird? *Anim. Behav.* 141, 183-194. 10.1016/j.anbehav.2018.05.018

[JEB244473C36] Munro, U., Munro, J. A., Phillips, J. B. and Wiltschko, W. (1997). Effect of wavelength of light and pulse magnetisation on different magnetoreception systems in a migratory bird. *Aust. J. Zool.* 45, 189-198. 10.1071/ZO96066

[JEB244473C37] Naef-Daenzer, B. (2007). An allometric function to fit leg-loop harnesses to terrestrial birds. *J. Avian Biol.* 38, 404-407. 10.1111/j.2007.0908-8857.03863.x

[JEB244473C38] Packmor, F., Klinner, T., Woodworth, B. K., Eikenaar, C. and Schmaljohann, H. (2020). Stopover departure decisions in songbirds: do long-distance migrants depart earlier and more independently of weather conditions than medium-distance migrants? *Mov. Ecol.* 8, 1-14. 10.1186/s40462-020-0193-1 32047634 PMC7006082

[JEB244473C39] Salewski, V., Bairlein, F. and Leisler, B. (2000). Recurrence of some palaearctic migrant passerine species in West Africa. *Ringing Migr.* 20, 29-30. 10.1080/03078698.2000.9674224

[JEB244473C40] Schmaljohann, H. and Eikenaar, C. (2017). How do energy stores and changes in these affect departure decisions by migratory birds? A critical view on stopover ecology studies and some future perspective. *J. Comp. Physiol. A* 203, 411-429. 10.1007/s00359-017-1166-8 28332031

[JEB244473C41] Schmaljohann, H. and Klinner, T. (2020). A quasi-experimental approach using telemetry to assess migration-strategy-specific differences in the decision-making processes at stopover. *BMC Ecol.* 20, 36. 10.1186/s12898-020-00307-5 32641125 PMC7346510

[JEB244473C42] Schmaljohann, H., Meier, C., Arlt, D., Bairlein, F., Van Oosten, H., Morbey, Y. E., Åkesson, S., Buchmann, M., Chernetsov, N. and Desaever, R. (2016). Proximate causes of avian protandry differ between subspecies with contrasting migration challenges. *Behav. Ecol.* 27, 321-331. 10.1093/beheco/arv160

[JEB244473C43] Schmaljohann, H., Eikenaar, C. and Sapir, N. (2022). Understanding the ecological and evolutionary function of stopover in migrating birds. *Biol. Rev*. 97, 1231-1252. 10.1111/brv.12839 35137518

[JEB244473C44] Skiles, D. D. (1985). The geomagnetic field its nature, history, and biological relevance. In *Magnetite Biomineralization and Magnetoreception in Organisms* (ed. J. L. Kirschvink), pp. 43-102. New York: Plenum Press.

[JEB244473C45] Svensson, L. (1992). *Identification Guide to European Passerines*. Stockholm: BTO.

[JEB244473C46] Taylor, P. D., Crewe, T., Mackenzie, S., Lepage, D., Aubry, Y., Crysler, Z., Finney, G., Francis, C., Guglielmo, C. and Hamilton, D. (2017). The Motus Wildlife Tracking System: a collaborative research network to enhance the understanding of wildlife movement. *Avian Conserv. Ecol.* 12, 8. 10.5751/ACE-00953-120108

[JEB244473C47] Wikelski, M., Arriero, E., Gagliardo, A., Holland, R. A., Huttunen, M. J., Juvaste, R., Mueller, I., Tertitski, G., Thorup, K. and Wild, M. (2015). True navigation in migrating gulls requires intact olfactory nerves. *Sci. Rep.* 5, 1-11. 10.1038/srep17061 PMC4657012 26597351

[JEB244473C48] Wiltschko, W. and Wiltschko, R. (1992). Migratory orientation: magnetic compass orientation of garden warblers (*Sylvia borin*) after a simulated crossing of the magnetic equator. *Ethology* 91, 70-74. 10.1111/j.1439-0310.1992.tb00851.x

[JEB244473C49] Wiltschko, W. and Wiltschko, R. (1995). Migratory orientation of European robins is affected by the wavelength of light as well as by a magnetic pulse. *J. Comp. Physiol. A* 177, 363-369. 10.1007/BF00192425

[JEB244473C50] Wiltschko, W., Munro, U., Beason, R. C., Ford, H. and Wiltschko, R. (1994). A magnetic pulse leads to a temporary deflection in the orientation of migratory birds. *Experientia* 50, 697-700. 10.1007/BF01952877

[JEB244473C51] Wiltschko, W., Munro, U., Ford, H. and Wiltschko, R. (1998). Effect of a magnetic pulse on the orientation of silvereyes, *Zosterops l. lateralis*, during spring migration. *J. Exp. Biol.* 201, 3257-3261. 9808838 10.1242/jeb.201.23.3257

[JEB244473C52] Wiltschko, W., Ford, H., Munro, U., Winklhofer, M. and Wiltschko, R. (2007). Magnetite-based magnetoreception: the effect of repeated pulsing on the orientation of migratory birds. *J. Comp. Physiol. A Neuroethol. Sens. Neural Behav. Physiol.* 193, 515-522. 10.1007/s00359-006-0207-5 17318656

[JEB244473C53] Wynn, J., Padget, O., Mouritsen, H., Morford, J., Jaggers, P. and Guilford, T. (2022). Magnetic stop signs signal a European songbird's arrival at the breeding site after migration. *Science* 375, 446-449. 35084979 10.1126/science.abj4210

