# Supplementary material for: A magnetic pulse does not affect free-flight navigation behaviour of a medium-distance songbird migrant in spring
Source: J Exp Biol. 2022 Oct 3;225(19):jeb244473. doi: 10.1242/jeb.244473 (PMC9604362; doi:10.1242/jeb.244473)
Supplement: Supplementary information [file jexbio-225-244473-s1.pdf]

# Supplementary Materials and Methods

## Analysis of radio tracking data

The data was automatically analysed with an R code written by the authors to identify the departure date, departure timing within the night, and the initial departure direction from Helgoland (Packmor et al. 2020; Müller, Rüppel, and Schmaljohann 2018; Müller et al. 2018). Further, the code analysed the arrival site at the shoreline along the German Bight to calculate the consistency of flight direction after departure from Helgoland. A person who did not participate in the field work and had no knowledge of which bird belonged to which of the two groups visually inspected the data for validity, and made changes wherever the algorithm did not detect the pattern correctly. A departure from Helgoland is generally characterised by a rapid increase in signal strength (bird's take-off) being detected by an increasing number of antennas (Fig. S2B), see also (Müller et al. 2018). We took this peak in signal strength as the bird's departure time within the night. When flying off the island, both the number of antennas detecting the signal and the signal strength decrease, indicating departure direction, before the signal is eventually lost (Fig S2B). Departure direction was defined as the weighted circular mean of the last half of the flight between take-off and last signal, similar to (Packmor et al. 2020; Müller, Rüppel, and Schmaljohann 2018; Müller et al. 2018). As the last detections often originated from just one antenna, departure directions often group according to the 22.5° resolution of the Helgoland radio-receiving system (Fig S2).

Based on those data, we defined the departure probability as a binary decision of the bird to stay or to depart during the first night after treatment, which was possible to assess for all of the 80 tagged birds. The exact departure timing, where the specific departure pattern was unambiguous (see paragraph above and Packmor et al. 2020; Müller, Rüppel, and Schmaljohann 2018; Müller et al. 2018), could be determined for 79 birds. Reliable departure direction could be assigned for 75 birds (for further details, see Packmor et al. 2020; Müller, Rüppel, and Schmaljohann 2018; Müller et al. 2018). We further analysed the consistency of the flight direction across the German Bight (Fig. 1B). The passage location on the shoreline was defined by the location of the radio-receiving station that recorded the first signal after a bird's departure from Helgoland, within the same night of the bird's departure from Helgoland. The passage at those receiving stations is generally characterised by an increase (bird is approaching the station) followed by a decrease (bird is flying away from the station) in signal strength over time, forming an inverse U-shape (Fig S2B). We defined the shoreline passage direction on the coast as the geographical angle between the location of the corresponding coastal radio-receiving station and the island of Helgoland (53°11′5.4″ N, 07°52′56.3″ E). Finally, we calculated the difference between the angle of departure from Helgoland and the shoreline passage direction. As such, 0° would represent no change in flight direction, e.g., a bird departing from Helgoland to the South and passing Wangerooge at the shoreline (53°47′ N, 07°55′ E; Fig. 1B). A change in flight direction of -90° would represent a bird departing to the East (90°) from Helgoland and passing Wangerooge in the same night. We could assign consistency in flight direction for 66 birds.

## R code

Click here to download R code

Journal of Experimental Biology • Supplementary information

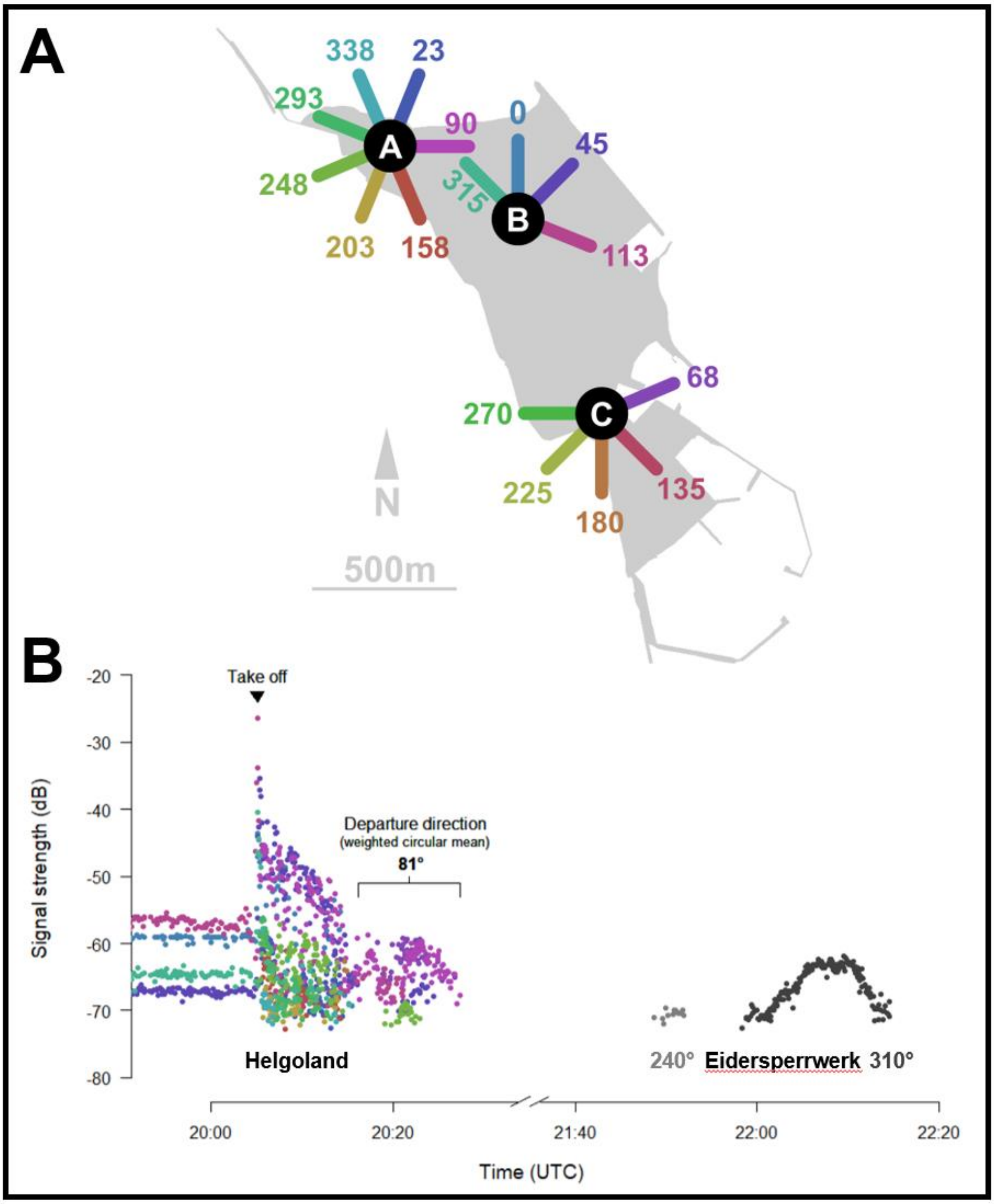


**Fig. S1. Radio telemetry. (A)** On Helgoland, three automated radio-receiving locations with 16 antennas, evenly spaced about 22.5° apart in direction, are installed to precisely determine departure timing and direction from Helgoland. **(B)** An example of a nocturnal departure event as recorded by our system displaying signal strength over time (Coordinated Universal Time: UTC). Colours correspond to antenna directions on Helgoland in the panel (A). The plot starts with four parallel lines, indicating the bird being stationary. The following peak of signal strength together with increasing numbers of different antennas (colours) indicates the take-off, i.e. the bird flying up. The following decreasing number of antennas and signal intensity indicate the bird flying off the island in a specific direction until the signal is lost. The grey dots after ~1¾ h indicate the passage at the shoreline radio-receiving station "Eidersperrwerk" (54.26N, 8.85E, ~63 km ENE of Helgoland).

Journal of Experimental Biology • Supplementary information

**Table S1.** Departure probability of European Robins (*Erithacus rubecula*) after treatment with a magnetic pulse and control group. Number of birds are given to the corresponding stopover duration after pulse application. Zero days represent birds, departing in the night after the application of the pulse.

| days to departure | control group (n=40) | treatment group (n=40) |
|---|---|---|
| 0 | 33 | 36 |
| 1 | 3* | 3 |
| 2 | - | - |
| 3 | 1 | - |
| 6 | - | 1 |
| 15 | 1 | - |
| 17 | 1 | - |
| 19 | 1 | - |

* one bird stayed at least one day, but its radio transmitter failed before leaving the island

**Table S2.**

Click here to download Table S2

**Supplemental References**

Müller, F., C. Eikenaar, Z.J. Crysler, P.D. Taylor, and H. Schmaljohann. 2018. 'Nocturnal departure timing in songbirds facing distinct migratory challenges', *Journal of Animal Ecology*, 87: 1102-15.

Müller, F., G. Rüppel, and H. Schmaljohann. 2018. 'Does the length of the night affect the timing of nocturnal departures in a migratory songbird?', *Animal Behavior*, 141: 183-94.

Packmor, F., T. Klinner, B.K. Woodworth, C. Eikenaar, and H. Schmaljohann. 2020. 'Departure decisions in songbirds following different migration strategies: Long-distance migrants depart earlier and more independently of weather conditions than medium-distance migrants', *Movement Ecology*, 8: 6.

Journal of Experimental Biology • Supplementary information
